# The Potential of MicroRNAs in Personalized Medicine against Cancers

**DOI:** 10.1155/2014/642916

**Published:** 2014-08-28

**Authors:** Anne Saumet, Anthony Mathelier, Charles-Henri Lecellier

**Affiliations:** ^1^Université Montpellier 1, 5 Bd Henri IV, 34967 Montpellier Cedex 2, France; ^2^Centre for Molecular Medicine and Therapeutics at the Child and Family Research Institute, Department of Medical Genetics, University of British Columbia, Vancouver, BC, Canada V5Z 4H4; ^3^Institut de Génétique Moléculaire de Montpellier, UMR 5535 CNRS, 1919 route de Mende, 34293 Montpellier Cedex 5, France; ^4^Université Montpellier 2, Place Eugène Bataillon, 34095 Montpellier Cedex 5, France

## Abstract

MicroRNAs orchestrate the expression of the genome and impact many, if not all, cellular processes. Their deregulation is thus often causative of human malignancies, including cancers. Numerous studies have implicated microRNAs in the different steps of tumorigenesis including initiation, progression, metastasis, and resistance to chemo/radiotherapies. Thus, microRNAs constitute appealing targets for novel anticancer therapeutic strategies aimed at restoring their expression or function. As microRNAs are present in a variety of human cancer types, microRNA profiles can be used as tumor-specific signatures to detect various cancers (diagnosis), to predict their outcome (prognosis), and to monitor their treatment (theranosis). In this review, we present the different aspects of microRNA biology that make them remarkable molecules in the emerging field of personalized medicine against cancers and provide several examples of their industrial exploitation.

## 1. Introduction

Recent technological advances in the field of molecular biology have revolutionized not only basic biological concepts but also clinical practice, in particular in the field of anticancer treatment. Management of patients with cancer is often based on the identification of tumor morphology, which decides the treatment program a patient should be enrolled in. However, pan-genomic analyses of genetic and epigenetic alterations and gene expression profiles are providing important new insights into the pathogenesis and molecular classification of cancers [1]. These rapidly diversifying and improving technologies to analyze tumors have revealed distinctive genomic (DNA mutations and chromosomal alterations), epigenomic (e.g., DNA methylation profiles), and transcriptomic (RNA expression profiles) differences between tumors that improve their classification in distinct molecular subtypes [2]. It is rapidly becoming apparent that each tumor has a unique combination of coding and noncoding mutations that distinguish between patients' tumors and therefore have the potential to serve as “signatures” in personalized anticancer therapies. It is possible to tailor patient medical care through the combination of individual genomic studies, phenotypic histomorphological features, and patient clinical specificities [3]. This approach, referred to as “personalized” or “individualized” medicine, is distinct from the classical “generalized” medicine as the medical decisions and selection of optimal therapies are not indiscriminately applied to each patient but rather take into account several parameters that identify the specific status of a patient. Personalized medicine will improve prediction of susceptibility to diseases and will restrict the development of cancers by anticipating disease progression. The use of personalized medicine will also reduce emergence of chemoresistance through the selection of drugs deemed most effective for each patient [2, 3]. This medical strategy will save time and improve cost effectiveness, not to mention significantly improving patients' quality of life by limiting the adverse effects of inappropriate treatments [2, 3].

One prerequisite for the development of personalized medicine is the identification of combinations of biomarkers to guide a physician's clinical decision. It is in this context that the potential of microRNAs (miRNAs), a particular class of small noncoding RNAs, has rapidly become apparent [4–6]. To date, more than two thousand human miRNAs have been identified [7]. These small RNAs orchestrate the expression of the genome at the posttranscriptional level and adapt the protein output to various intracellular or extracellular stimuli. As such, they impact many, if not all, cellular processes and their deregulation is causative of many human malignancies, including cancers [8–11]. A plethora of researchers have now implicated miRNAs in the initiation and progression of primary tumors, as well as in metastasis formation [12–14]. More than 12,600 publications related to miRNA and cancer are listed in the NCBI PubMed database and their number exponentially grows. Advantageously, cancer cell types tend to have a highly specific cellular repertoire of miRNAs [15–18]. The expression levels of miRNA can be monitored in a variety of human specimens, including fresh or formalin-fixed paraffin embedded (FFPE) tissues [19, 20], as well as in almost all human body fluids [21–25]. Moreover, recent studies revealed that specific miRNA expression levels in biological fluids are associated with chemotherapy responses [23, 26, 27]. Hence, in addition to their potential as targets of novel anticancer therapies, several aspects of miRNA biology make them excellent candidates as biomarkers to be used in innovative and noninvasive tests aimed at identifying various cancers (diagnosis), predicting their outcome (prognosis), and monitoring their treatments (theranosis) [21, 25, 28–31]. Here we review the different aspects of miRNA biology that establish their potential in the emerging field of personalized medicine against cancers. We also present several known limitations of their exploitation, as well as future challenges and ongoing industrial developments.

## 2. miRNA Biogenesis and Mechanism of Action

miRNAs are noncoding RNAs, typically ~18–22 nucleotides long, which are generated through a complex multistep process. Several excellent reviews have already thoroughly described this process (see [32–35]). We focus here on the events required for the understanding of this review. MiRNA genes are first transcribed by RNA polymerase II into long, capped, and polyadenylated primary miRNA precursors (pri-miRNAs). The pri-miRNAs are subsequently processed by the nuclear RNase III enzyme Drosha into precursor miRNAs (pre-miRNAs) [36–38]. The pre-miRNAs are exported from the nucleus to the cytoplasm where they are cleaved by the cytoplasmic RNase III enzyme Dicer into a double-stranded RNA duplex (miR-5p/miR-3p). Alternative pathways exist that bypass either the Drosha or the Dicer steps [39], but these pathways invariably produce a miR-5p/miR-3p duplex. The two strands of the duplex are then incorporated into the effector complex called the miRNP complex, which contains several proteins including the key Argonaute proteins. One strand of the miRNA duplex then redirects the miRNP onto RNAs that harbor partial sequence complementarity. The study of the mechanisms responsible for the recognition of RNAs by miRNAs is an intense field of research with rapidly evolving concepts (e.g., location of miRNA binding sites [40, 41]). Canonical models are based on imperfect base-pairing between the mature miRNA and the targeted RNA. The extent to which the 5′ end of the mature miRNA (referred to as the “seed”) pairs with the targeted RNA is of particular importance for the efficacy of miRNA-target interactions [42]. The miRNP complex eventually induces mRNA degradation and/or repression of translation [43, 44]. While the former's mechanism relies on deadenylation and further exonucleolytic cleavage of the mRNA, the latter's remains unclear and is debated as translation repression could occur at different steps: inhibition of initiation, inhibition of elongation, cotranslational protein degradation, or premature termination of translation [43].

The expression of miRNAs is a tightly regulated process that is extremely sensitive to intra- and extracellular stimuli (e.g., hormones, vitamins, pharmacological molecules, or hypoxia) [13, 45–49]. As a consequence, each cell type, at a particular time and a particular location, harbors a particular miRNA repertoire. This important concept constitutes the basis of the remarkable interest in miRNAs within the field of oncology. The potential importance of miRNAs to medicine was first highlighted by the seminal findings of Chen et al. [50] who demonstrated that some miRNAs are expressed in hematopoietic cells and showed that their expression was dynamically regulated during early hematopoiesis and lineage commitment. Importantly, they showed that miR-181 was preferentially expressed in the B-lymphoid cells and that its ectopic expression in hematopoietic stem/progenitor cells led to an increased fraction of B-lineage cells. Thus, it was illustrated that it is possible to distinguish different cell types or different cellular conditions (i.e., treatment) based on miRNA profiling. Moreover, miRNA expression levels in somatic cells of male and female patients can differ, likely due to exposure to specific hormones (e.g., testosterone, estrogen, and androgen), an observation that can explain gender-related differences noted in disease outcome and pathogenesis [51, 52]. Similarly, the expression of some miRNAs can be linked to aging [53–55]. The specificity of the cellular miRNA repertoire and its sensitivity to a large panel of intra/extracellular stimuli and characteristics (including gender and age) have stimulated interest not only in basic research focused on deciphering the contribution of miRNAs to cancer development, but also in more applied research aimed at evaluating miRNAs' potential in cancer personalized medicine.

## 3. miRNAs and Cancer

Extensive research has shown that miRNAs play essential roles in cancer initiation, progression, and metastasis formation [56–59]. The miRNA expression levels in tumors can be up- or downregulated compared to normal tissue, and several miRNAs have been directly implicated in tumorigenesis by acting either as “oncomirs” or tumor suppressor miRNAs [15, 60, 61]. Among them, we can cite the miR-17–92 cluster (several miRNAs transcribed in a single transcription unit/pri-miRNA), which was the first oncogenic miRNA locus described [62]. Conversely, the miR-34a is an important miRNA with tumor suppressor activity, which can be directly transactivated by p53 [60]. Its upregulation results in increased apoptosis and altered expression of genes related to cell cycle progression, apoptosis, and angiogenesis [63]. As observed for protein coding genes [64–68], individual miRNAs can behave as oncogenes in one cell type and as tumor suppressors in others [69, 70]. For example, miR-221 acts as an oncogene in liver cancer by downregulating the expression of the tumor suppress or phosphatase and tensin homolog (PTEN), but it acts as a tumor suppressor in erythroblastic leukaemia by reducing the expression of the KIT oncogene [69, 70]. This dual action can be attributed to specific cellular contexts which expose a miRNA to distinct transcriptional regulation and/or to different RNA targets [42, 71].

The changes in the miRNA repertoire observed in cancer can result from (1) various disruptive mechanisms occurring at genes (deletions, amplifications, or mutations of miRNA genes), (2) regulation of transcription (epigenetic silencing, deregulation of transcription factors), or (3) posttranscriptional regulation (deregulation of the miRNA biogenesis pathway) [13, 72, 73]. One of the first implications of miRNAs in cancer was the discovery that the gene encoding miR-15a and miR-16 is frequently deleted in chronic lymphocytic leukemia [12]. This observation was further supported by other miRNA genes in other types of cancers [74–76]. The transcriptional deregulation of miRNA genes is mechanistically similar to what is observed in the case of coding genes and relies on similar processes (DNA methylation, histone acetylation, defect in specific transcription factor binding) [77, 78]. We have, for instance, demonstrated that the PML-RARA oncogenic protein associated with acute promyelocytic leukemia represses retinoic acid-responsive miRNA genes similar to coding genes [79]. Likewise, in breast cancer cells, the antagonism between RARA and ESR1 initially observed in the case of coding genes [80] also occurs on miRNA genes [45]. The deregulation observed at the posttranscriptional level (i.e., biogenesis of miRNA) is manifestly more specific to miRNAs. For instance, the LIN28 protein, a developmentally regulated RNA binding protein, whose expression is reactivated in many human tumors, can specifically block the Drosha cleavage of the pri-miRNAs belonging to the let-7 family [81]. The expression of several proteins (e.g., Dicer, Drosha, and Argonaute 2) involved in the biogenesis, processing, or the action of the miRNAs can be perturbed in certain cancers with presumably even more broad impact on cell physiology [82, 83]. The combinatorics of varied sources of deregulation generates miRNA profiles are specific to cancer types/subtypes and are often associated with staging, progression, and response to chemotherapies [15–18, 26, 60, 84, 85], thereby providing a means for the development of miRNA-based diagnostic, prognostic, and/or theranostic tests.

## 4. miRNA and miRNA Target Site Alterations in Cancer

Alteration of miRNA-mediated posttranscriptional regulation can be the consequence of genomic variations specific to cancer. Studies have shown that genomic mutations observed in cancer cells can drastically perturb miRNA-mediated regulation by modifying either the sequence of the miRNAs or the sequence of their targets. Intensive efforts are developed to collect the relevant data and to develop tools for their analysis. The first studies assessing the impact of mutations on miRNA-mediated regulation focused on polymorphic mutations (single nucleotide polymorphism (SNP)) (see [86] for a review). Bioinformatics studies highlighted SNPs in cancer samples located in pri-miRNAs, pre-miRNA, mature miRNAs, and miRNA targets with a potential impact on miRNA biogenesis, or the process of miRNA-mediated posttranscriptional regulation [87–90]. Operating under the knowledge that mRNAs are predominantly targeted by miRNAs in their 3′UTRs [91], Bruno et al. used SNP data to create the miRdSNP database, which stores disease-associated SNPs located in the 3′UTRs of genes and are supported by the literature after manual curation of publications stored in PubMed [92]. The most recent version of miRdSNP (v.11.03) stores 175,351 SNPs in 3′UTRs with 630 disease-associated SNPs for 204 diseases (including ~30 cancers). While previous studies have mainly been focused on SNPs, an increasing number of studies provide access to patient-specific somatic SNVs. Last year, Bhattacharya et al. created SomamiR [93], the first comprehensive database of mutations from whole-genome sequencing of cancer samples obtained by extracting mutations specific to cancer samples when compared to matched normal samples. The database provides the community with germline and somatic mutations in miRNAs and target sites that have the potential to functionally altering miRNA regulation. Importantly, the database stores experimental information about the impact of the mutations on miRNA function and their association with cancer.

Bioinformatics analyses of SNVs in miRNA target sites are critical for predicting functionally impactful mutations, but the development of bioinformatics approaches has only recently started to be the focus of concerted genomewide efforts. By combining whole-genome sequencing data from The Cancer Genome Atlas pan-cancer data set with Argonaute crosslink immunoprecipitation (AGO-CLIP) data, Hamilton et al. [94] defined a set of miRNA target sites derived from AGO-CLIP that were mutated in cancer. The algorithm developed by Hamilton et al. was then used to identify thousands of SNVs in miRNA binding sites. By combining these datasets with mRNA expression, they highlighted expression changes correlating with mutations. Four out of six tested mutations successfully exhibited experimentally strong evidence of miRNA binding and regulation. An alternative approach for highlighting mutations that impact miRNA regulation is through analyses of the transcriptome. In [95], the authors identified 73,717 SNVs in UTRs from transcriptome data of non-small-cell lung cancer samples. This set of SNVs was processed to predict mutations affecting miRNA secondary structure and target sites. The computational analysis highlighted 490 SNVs with potential effects on miRNA target sites; the SNVs in turn are associated with genes enriched in molecular mechanisms of cancer. In the recent years, it has become apparent that mutations in miRNAs and miRNA target sites that play a critical role in cancer development. As an increasing number of cancer whole-genome sequence datasets will become publicly available to the community in the near future, it is critical to develop dedicated computational tools for the identification of mutations altering miRNA-mediated regulation. This will enable the community to better understand the underlying causes of carcinogenesis at the level of miRNA regulation and promises to significantly contribute to the vision of personalized diagnosis and therapeutic treatment.

## 5. miRNA-Based Cancer Diagnosis

While cancer-specific mutations in miRNA genes and/or their targets can be detected by classical DNA sequencing technologies, miRNA expression profiling requires more specific approaches. Three types of miRNA profiling technologies are currently used: RT-qPCR, microarrays, and RNA sequencing. The RT-qPCR approach requires a particular reverse transcription step which is primed by a stem-loop oligonucleotide [96]. This primer can pair with the 3′ region of the mature miRNA or with an adapter than have been ligated to its 3′ end. This latter solution allows the use of one single RT primer while adding a ligation step. The PCR step can rely on either the Taqman or SYBRGreen technology. The RT-qPCR does not necessitate large amounts of RNA and is traditionally recognized as highly sensitive and specific. Several assays are commercially available either in a specific single miRNA format or as arrays that can correspond to hundreds of miRNAs (this number is limited by the plates used for qPCR). Conversely, microarrays can detect more miRNAs in one single experiment, but this approach is considered to be less specific. Both RT-qPCR and microarrays are targeted technologies that do not allow the detection of novel miRNAs that are constantly identified [97, 98]. As an alternative, RNA sequencing is obviously the most powerful profiling technology in terms of both specificity and sensibility, but its cost is still high (compared to RT-qPCR or microarray) and the data generated require substantial computational processing.

In addition to intracellular expression, miRNAs can be detected in extracellular compartments. The presence of specific extracellular and circulating miRNAs in several body fluids of cancer patients is now largely described [21, 22, 25]. These circulating miRNAs are particularly interesting in the context of personalized medicine because correlations between high levels of specific circulating miRNAs and the response to a given anticancer treatment have been observed [26, 88, 99]. For instance, levels of miR-21 were found elevated in the serum of patients suffering from metastatic hormone-refractory prostate cancer especially in those patients resistant to docetaxel-based chemotherapy [27]. Studies in gastric and bladder cancers also identified specific miRNAs involved in cisplatin resistance [100–102]. Although the molecular basis behind the secretion of miRNAs remains largely unknown, it appears to be specific. The secretion of miRNAs represents a potent mode of intercellular communication that can, for instance, create a favorable context for the implantation of metastasis and the formation of secondary tumors [99, 103]. The miRNAs circulating in body fluids also present the remarkable characteristic of being extremely stable, though the mechanistic basis of this resistance to degradation remains largely unclear [104]. One reason could lie in the fact that circulating miRNAs are packaged in exosomes or other microvesicles present in body fluids as well as associated with (lipo)proteins (HDL and Argonaute 2) [105–107].

Similar to mutations, several miRNA profiles in various human specimens and cancers have been collected and made publicly available in dedicated databases: PhenomiR [108], oncomiRDB [61], PROGmiR [109], miRò [110], and miRandola [111]. We acknowledge that revisiting these data can dampen enthusiasm for the diagnostic/prognostic potential of miRNAs [112, 113]. In fact, no matter the technology used or the tissue studied, several issues associated with standardization of samples manipulation, miRNAs extraction protocols, measurements, and statistical analyses still require improvements [114–116]. Several papers have previously tackled the importance of samples processing [117, 118]. For example, hemolysis occurring during blood collection can have significant impact on miRNA profiling in plasma/serum [119–122]. The evaluation of the quantity and quality of miRNAs isolated from biological samples is indeed a key step in miRNA profiling. Although methods for miRNA extraction are usually similar to that used in the case of total RNAs (with possibly only slight modifications required to retain the small RNA fraction), the sizes and relative abundance of ribosomal RNAs cannot give information about the integrity of the miRNA preparation. In addition, the quantification of miRNA preparations can only be accurate in samples where larger RNAs are not degraded as the degradation products can compromise this quantification. Moreover, the low concentration of RNAs present in certain body fluids makes the estimation of miRNAs abundance particularly difficult [123]. The measurement of miRNA expression can also be affected by certain compounds coextracted with RNAs [124]. Strikingly, it has been reported that short RNAs with low GC content may be selectively lost during extraction depending on the extraction methods [125]. In addition to these experimental steps, data standardization and normalization as well as the evaluation of their statistical significance must also be carefully defined.

Overall, it is likely that inconsistencies in any of the steps described above will impede the definition of robust cancer-specific miRNA signatures [112, 113], but a better definition and standardization of the protocols used will undoubtedly overcome these obstacles [104, 126]. Several companies have indeed decided to meet the challenge (e.g., Santaris Pharma, Rosetta Genomics, Cepheid, Prestizia-Theradiag, and IntegraGen [127]). These efforts have, for instance, revealed that the expression level of the miR-31-3p allows the identification of patients with wild-type KRAS metastatic colorectal cancer responding to anti-EGFR therapy [128]. With approximately two-thirds of metastatic colorectal cancer patients being wild-type KRAS, this marker could help better use EGFR therapy and spare patients from inappropriate treatment.

## 6. miRNA-Based Anticancer Therapy

It is important to note that miRNA deregulations observed in cancers are not necessarily involved in carcinogenesis, but that such deregulation could constitute potent biomarkers nonetheless. In contrast, some miRNAs have been truly functionally implicated in the development/progression of cancer or in the integration of chemotherapies. In that specific case, miRNAs can represent appealing candidate targets for novel anticancer therapies [129–131]. In fact, some pharmaceutical companies are already finalizing preclinical research phases and proceeding to clinical trials (see below). In addition to pharmacological agents classically used in oncology and able to control transcription and miRNA expression [132, 133], two miRNA-specific technological approaches can be envisaged: (i) to downregulate or block the function of oncogenic miRNAs (miRNA antagonists) and (ii) to upregulate the expression of miRNAs that have a tumor-suppressive function (miRNA mimics). The ultimate goal of these manipulations would be to restore a nonpathogenic miRNA profile [129–131] but, even more interesting in the context of personalized medicine, they can also sensitize cancerous cells to a particular chemo/radiotherapy. In fact, some miRNAs are implicated in the integration of drug effect [100–102] and modulating these miRNAs would restore the sensitivity of drug-resistant cells to chemotherapy and would prevent tumor recurrence [134, 135], as exemplified in the case of microRNA-200c [136].

Current strategies to inhibit miRNAs are mainly based on antisense oligonucleotides (also known as anti-miRs including locked nucleic acids (LNA anti-miRs), tiny LNA anti-miRs, and antagomirs) which titer the targeted miRNA [137–140]. They usually involve the introduction of a chemically modified single stranded RNA that binds with high affinity to a miRNA of interest. Since pairing with the inhibitor is very stable, the targeted miRNA is unable to repress translation. LNA-mediated miRNA silencing was shown to be efficient* in vivo* even in non-human primates [141]. In fact, an LNA-based inhibitor of miR-122, miravirsen, is currently being tested in phase 2 clinical trials for the treatment of hepatitis C virus infection [142]. Another strategy used to inhibit miRNA is to introduce within the cells an artificial RNA decoy, also called miRNA sponge, which harbors several binding sites complementary to a miRNA of interest [143–145]. This miRNA sponge can be produced from a transgene allowing stable expression even* in vivo* [143]. It is interesting to note that this artificial strategy is in fact an endogenous regulatory process, which involves long non-coding RNAs, referred to as competing endogenous RNAs (ceRNAs), acting as miRNA sponges [146]. Whether this situation is widely encountered and could occur with different long noncoding RNAs is still debated [147], but similar strategies have also been described in the case herpesvirus saimiri, which produces an RNA decoy able to titer the miR-27 [148]. In addition to nucleic-acid based strategies (i.e., anti-miRs and miRNA sponges), small chemical molecules able to block the processing of the pre-miRNAs by Dicer are also envisaged [149].

On the other hand, artificial restoration of the expression or function of one or a limited number of miRNAs, also called “miRNA replacement therapy,” can be achieved either with miRNA mimics (typically introduced in the cell as pre-miRNAs) or with miRNAs directly encoded by expression vectors. In many cases, the reintroduction of these miRNAs leads to a reactivation of pathways that are required for normal cellular function [150, 151]. It is worth mentioning that a clinical trial using a miR-34 mimic is already in progress [152, 153]. In preclinical studies, it was reported that the injection of miR-34a mimic extended the survival of tumor-bearing mice [58]. Another study demonstrated that systemic administration of a miR-34 in a pancreatic xenograft cancer model significantly inhibited tumor growth and induced cancer cell apoptosis [154]. In May 2013, the Mirna Therapeutics Company initiated a phase I study to evaluate the safety of MRX34, a liposome-formulated mimic of miR-34, in patients with unresectable primary liver cancer and advanced or metastatic cancer (ClinicalTrials.gov Identifier: NCT01829971). Likewise let-7 mimics are in preclinical development stages at Mirna Therapeutics.

In addition to these chemical and synthetic procedures, miRNA expression levels can also be adjusted through dietary manipulations. Several nutrients such as amino acids, carbohydrates, fatty acids, vitamins, and phytochemicals (curcumin, resveratrol) are indeed known to modulate miRNA expression levels [155, 156]. For instance, intake of dietary fiber is inversely associated with colorectal cancer risk [157]. The microbial anaerobic fermentation of dietary fiber produces short chain fatty acids (such as acetate, propionate, and butyrate) and butyrate, whose bioavailability is reduced in case of low fiber intake, was shown to decrease the expression of several oncogenic miRNAs in HCT-116 (miRs-17, -20a, -20b, -93, -106a, and -106b) [158]. Hence, although further studies are required to fully unveil the mechanisms underlying diet-mediated miRNA regulations, modulating food intake may contribute to novel miRNA-based anticancer strategies that could be easily adapted to patient's requirements.

## 7. Conclusion

The discovery of miRNAs, and their implication in cancer, has not only intensified the “noncoding RNA revolution” [159] but also opened up new prospects in biomarker and therapeutic target studies [26, 27]. These molecules harbor specific features (stability, easy manipulation, reasonably simple detection, and tissue specificity) that can guide individualized treatments and monitoring of cancers. Some limits still exist that may prevent their immediate large-scale exploitation, but collective efforts currently made by both academic and industrial researchers will certainly circumvent these constraints and rapidly transfer miRNAs from bench to bedside. We also anticipate that this particular field of research, and the field of personalized medicine as a whole, will encourage (not to say demand) the acquisition of novel expertise and competences by physicians in order to understand and combine computational/experimental biology together with medical practices.
